# Automated Lung Cancer Detection Using Artificial Intelligence (AI) Deep Convolutional Neural Networks: A Narrative Literature Review

**DOI:** 10.7759/cureus.10017

**Published:** 2020-08-25

**Authors:** Kaviya Sathyakumar, Michael Munoz, Jaikaran Singh, Nowair Hussain, Benson A Babu

**Affiliations:** 1 Family Medicine, University of Florida College of Medicine, Gainesville, USA; 2 Pediatrics, Monmouth Medical Center, Long Branch, USA; 3 Internal Medicine, Saint John Regional Hospital, Saint John, CAN; 4 Internal Medicine, Ross University School of Medicine, Bridgetown, BRB; 5 Hospital Medicine, Northwell Health, New York, USA

**Keywords:** lung cancer, computer-aided detection, lung nodule, convolutional neural networks, deep neural network, ensemble neural network, artificial intelligence, deep learning artificial intelligence

## Abstract

Lung cancer is the number one cause of cancer-related deaths in the United States as well as worldwide. Radiologists and physicians experience heavy daily workloads, thus are at high risk for burn-out. To alleviate this burden, this narrative literature review compares the performance of four different artificial intelligence (AI) models in lung nodule cancer detection, as well as their performance to physicians/radiologists reading accuracy.

A total of 648 articles were selected by two experienced physicians with over 10 years of experience in the fields of pulmonary critical care, and hospital medicine. The data bases used to search and select the articles are PubMed/MEDLINE, EMBASE, Cochrane library, Google Scholar, Web of science, IEEEXplore, and DBLP. The articles selected range from the years between 2008 and 2019. Four out of 648 articles were selected using the following inclusion criteria: 1) 18-65 years old, 2) CT chest scans, 2) lung nodule, 3) lung cancer, 3) deep learning, 4) ensemble and 5) classic methods. The exclusion criteria used in this narrative review include: 1) age greater than 65 years old, 2) positron emission tomography (PET) hybrid scans, 3) chest X-ray (CXR) and 4) genomics. The model performance outcomes metrics are measured and evaluated in sensitivity, specificity, accuracy, receiver operator characteristic (ROC) curve, and the area under the curve (AUC).

This hybrid deep-learning model is a state-of-the-art architecture, with high-performance accuracy and low false-positive results. Future studies, comparing each model accuracy at depth is key. Automated physician-assist systems as this model in this review article help preserve a quality doctor-patient relationship.

## Introduction and background

Lung cancer is the number one cause of cancer-related deaths in the United States and worldwide [1]. Furthermore, lung cancer has amongst the highest public burden of cost worldwide. Healthcare costs to Medicare beneficiaries were analyzed: the highest costs were related to surgery and an estimated $30,000 over a 15-year period [2]. Similarly, patients receiving chemotherapy and radiation therapy faced a cost of $4000-$8000 per month, with an average life expectancy of 14 months from the time of diagnosis [2]. Europe’s incidence of lung cancer is estimated to be 60 per 100,000 inhabitants. Its costs of healthcare and management for the patient post-intervention are estimated to be 17,000 Euros per year [3].

The National Lung Screening Trial (NLST) found that examination with low-dose computed tomography (LDCT) instead of the standard chest X-ray, in a high-risk population, led to a 20% reduction in mortality rate [4]. Additionally, the detection rate of lung cancer screening with low-dose CT is 2.6- to 10-fold higher than that with chest radiography [5]. The key to reducing lung-cancer-related deaths is early diagnosis and this relies on fast and accurate detection of lung nodules and careful examination of chest CT scans to determine malignancy - a process which requires considerable time and effort on behalf of radiologists and physicians.

According to a recent study, physicians spend 75% of each patient visit on activities other than face-to-face patient encounter, including working with the electronic medical records (EMR) [6]. Studies also found that physicians from various specialties spend up to 2 hours on administrative duties for each hour that they see patients in the office, followed by an additional 1 to 2 hours of work after clinic, mostly devoted to the EMR [7]. It is likely, although not investigated, that these figures are much higher for physicians screening patients at risk for lung-cancer, due to the time required for the initial examination and evaluation of CT scans.

During the 18th World Conference on Lung Cancer (WCLC), Dr Flanou confirmed that oncologists were at highest risk from burn-out compared to other physicians as well as other oncology care staff (nurses, psychologists and social workers), with a reported prevalence between 35-60%. Amongst individuals who suffer burn-out there is a risk of mental health issues in 20-35%, moreover in physicians it is associated with a decrease in empathy towards patients and reduced quality of care [8]. It is therefore of utmost importance that all ways in which the burden of work on physicians may be reduced, should be explored, for the wellbeing of both the patients and physicians.

One such solution is artificial intelligence (AI) automated CT lung cancer detection, which can be used to assist physicians, thereby reducing their burden of work, optimizing hospital operational workflow, and providing more time to develop a high-quality doctor-patient relationship. A computer-aided detection (CAD) system was first introduced by Niki et al. as a means to extract and analyze data from CT scans, classify benign and malignant lung cancer changes, and for the purpose of screening patients using 3D CT scans [9]. Since then, numerous studies have found improved detection of lung nodules on CT scans when examination by a physician/radiologist is combined with the use of a CAD system [5,10]. Improved radiologist performance with CAD was noted especially in the detection of small lung nodules, <5 mm in size, which are often easily overlooked by visual inspection alone [11]. Thus, CAD and its associated AI models help not only to reduce the burden of work on physicians, and subsequently fatigue-related errors of judgement, but to improve detection of nodules particularly in the early stages of lung cancer, which are more likely to be missed.

Methods

Method Outline

PICO framework, a universally accepted standard within the research community that stands for the acronym PROBLEM: in this narrative review looks at lung cancer, INTERVENTION: this review also looks deeper into the artificial intelligence (AI) application comparing each research team's AI deep learning model performance, COMPARISON: this review also explores the deep learning ensemble convolutional neural network method (CNN) and compares with the four research groups' classic machine learning classifier performance. AI model performance OUTCOMES are measured for each research team model performance sensitivity measuring how well the algorithm recognizes the type of lung nodule correctly. Model specificity measures the ability of the algorithm to remove the false positives, and a high specificity value means a low rate of misdiagnosis of lung cancer. The model accuracy measures the proportion of data that was classified correctly. Lastly, both receiver operator characteristic (ROC) curve and area under the curve (AUC) were used in the various research group model performance metrics.

A total of 648 articles were selected by two experienced independent physicians in the fields of pulmonary critical care, hospital medicine. Physician reviewers selected four out of 648 studies. Each article selected from the years ranging between 2008 and 2019.

The narrative review inclusion criteria are: 1) 18-65 years old, 2) CT chest scans, 3) lung nodule, 4) lung cancer, 5) deep learning, 6) ensemble, and 7) classic machine learning classifier methods. This literature review exclusion criteria include the following: 1) age greater than 65 years old, 2) PET hybrid scans, 3) chest radiographs, and 4) genomics.

The databases searched, selected in this narrative review use a rigorous selection criteria from the following databases: PubMed/MEDLINE, EMBASE, Cochrane Library, Google Scholar, Web of Science, IEEEXplore, and DBLP. The search word terms used a combined systematic methodology consisting of truncated terms, wild card, Boolean and fuzzy logic methods.

Narrative Literature Review of the Model Research Experiment Done by Toğaçar et al.

Toğaçar et al. proposed a hybrid model of the combined LeNet, AlexNet, and VGG-16 [12,13]. Then, the features from this hybrid model, the last fully-connected layer of convolutional neural networks (CNNs), were used as an input for the following machine learning/classification models: linear regression (LR), linear discriminate analysis (LDA), decision tree (DT), support vector machine (SVM), k-nearest neighbor (kNN) and softmax function. These machine learning classifiers were then tested and each model performance metric was compared amongst each other. To improve the model classification accuracy, image augmentation techniques were conducted while training the models. Twenty additional images were obtained from the dataset. Then, the minimum redundancy maximum relevance (mRMR) feature selection method identified the efficient features, that are then used as the input to this hybrid model.

Their architecture consists of convolutional and average pool layers [12,13]. It further consists of a straightener convolutional layer, two fully connected layers and a softmax classifier. LeNet includes a 5 x 5 filter. The size of the images varies from 32 x 32 x 1 to 28 x 28 x 6. The Alexnet architecture is made up of ﬁve convolutional, three pooling and three fully connected layers. Their convolutional layer is made up of a circulating ﬁlter over the entire image. Filters vary in sizes ranging from 3 x 3, 5 x 5 and 11 x 11 dimensions.

The VGG-16 architecture is made up of a 16-layer network structure. An important feature of this architecture is an increased network structure. The size of the input layer was 224 x 224; the ﬁlter size was 3 x 3. Their architecture was composed of ﬁve convolutional layers, a pooling layer, and three fully connected layers. Lastly, they used the softmax in the classiﬁcation of tasks.

**Figure 1:**
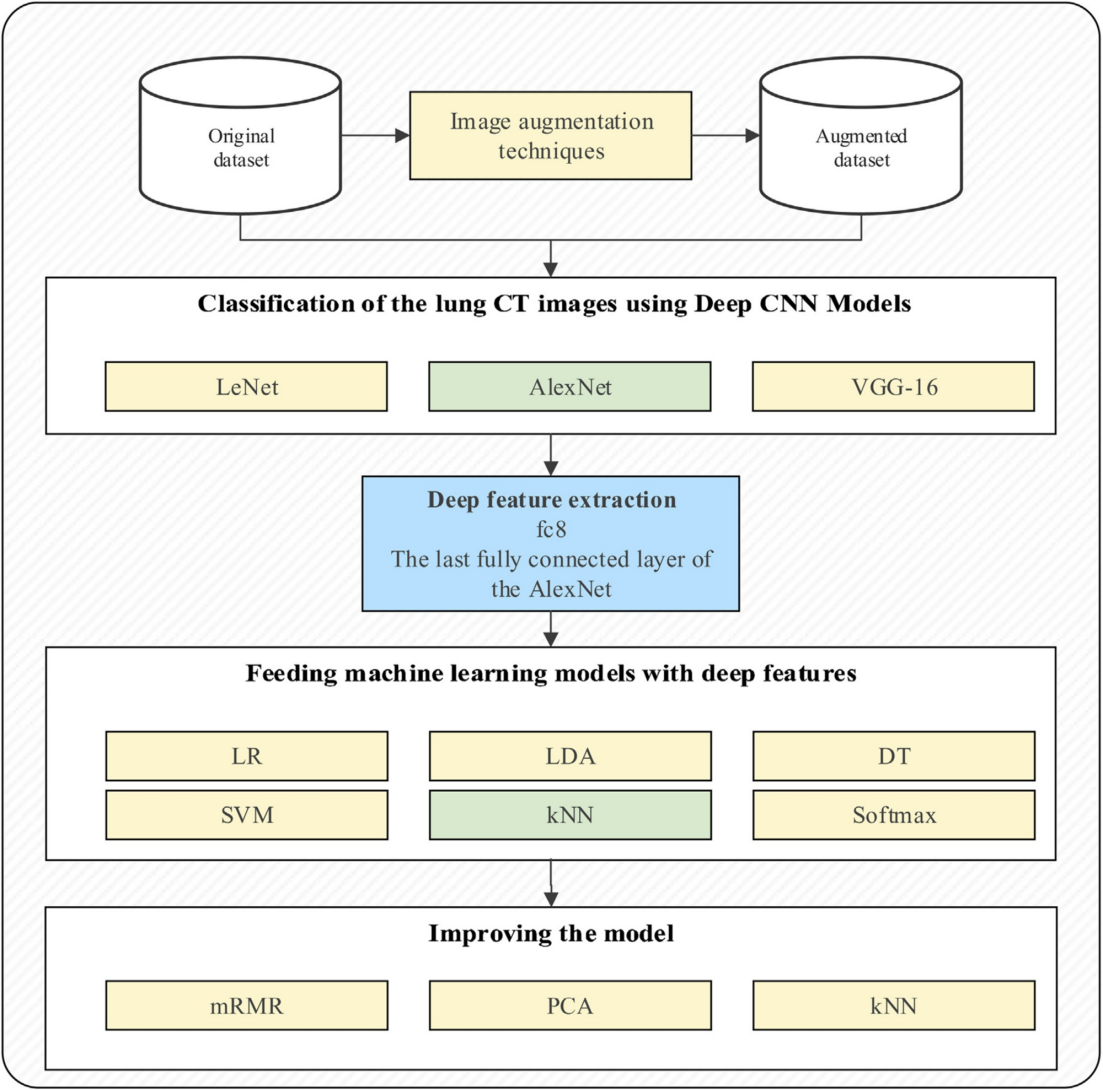
Flowchart of Model Permission for reprint obtained from Toğaçar et al. [13]

Toğaçar et al. used both well-known Adam and RMSProp optimization methods for the LeNet architecture. Both the AlexNet and VGG-16 architectures used the classic stochastic gradient descent method for optimization. Figure 1 below shows their basic architecture of the ensemble models described above.

## Review

Results and discussion

*The Model Performance Analysis*

Minimum redundancy and maximum relevance (mRMR) feature selection method with ensemble CNN performed better than the methods described in the three other papers. It is the use of additional techniques such as image augmentation, principal component analysis (PCA), mRMR and appropriate feature selection that made this performance difference [13].

In the last iterations, the dimensions of the feature set obtained using image augmentation techniques were reduced using PCA before the classification task [13]. Then, this reduced feature set was the input to the KNN classifier, with an accuracy of 97.92%. Then, the KNN classifier was used with the input mRMR algorithm with the 1000 features [13]. These features were obtained from the fc8 layer of AlexNet architecture. Thirty-three, 50, 100, 150 and 200 of the most efficient features were determined and ranked, respectively [13]. The extracted features were then reclassified with KNN. A 10-fold cross-validation method was used for further model performance testing [13].

PCA method decreased the model accuracy from 98.74% to 97.92% [13]. This method obtained this level of success with only 33 features; it took up less time during model training. This method time saved is because fewer features were used. In addition, the model performance metric results of the KNN classifier with and without PCA method were very close [13].

Second, 1000 efficient features were selected by the mRMR method, obtained from the last layer of AlexNet without using the PCA method [13}. Success obtained was 99.51% with 200 features provided by mRMR. Hundred, 150 or 200 features from the mRMR algorithm were more successful than using all 1000 features obtained from the fc8 layer of AlexNet [13].

**Figure 2:**
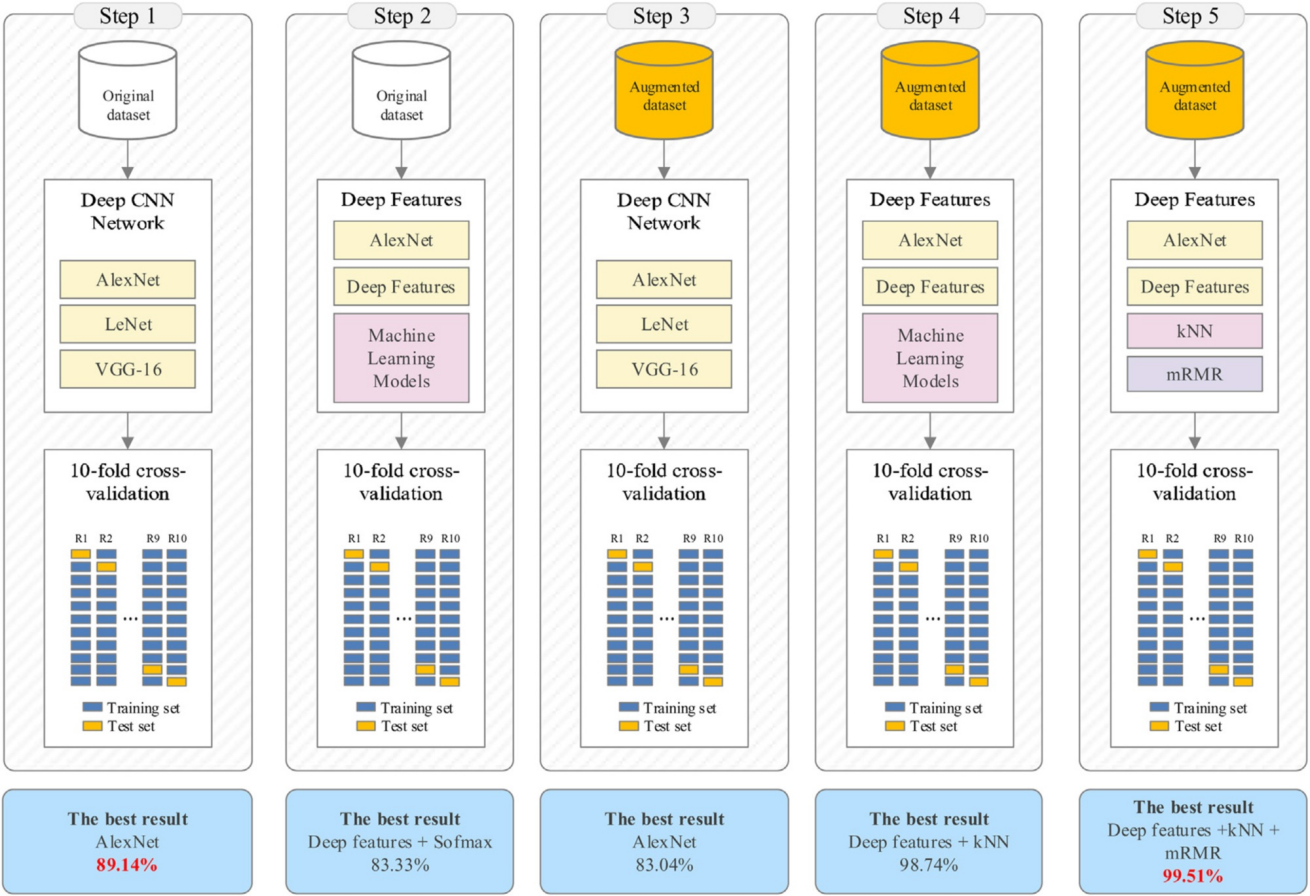
Flowcharts showing the various iterations and corresponding performance metrics Permission for reprint obtained from Toğaçar et al. [13]

Toğaçar et al. group then extended the testing by focusing on the KNN classifier method [13]. The k-value corresponding to the number of the nearest neighbors was searched between 100 and 102 considering various distance functions by using the Bayesian optimization method. The classification success decreased relatively and the k-value increased [13]. Efficient results were ensured for KNN when the k was set to 1 and the distance function was adjusted to the correlation. These researchers then performed the 10-fold cross-validation for model evaluation. Toğaçar et al. model finally achieved an accuracy of 99.51%, sensitivity of 99.32%, specificity of 99.71% and F-score of 99.51% (Figure 2) [13].

Literature on Performance Comparisons

In a different research team model comparison da Silva et al. [12,14] in their convolutional neural network-based particle swarm optimization (PSO) for lung nodule achieved a lower accuracy of 97.62% in comparison to the Toğaçar et al. model, mainly because no image augmentation and feature selection technique was used before the CNN architecture, whereas Toğaçar et al. used the mRMR technique to achieve a model accuracy of 99.51% [13].

In another research group Jung et al. [12,15] model performs lower in accuracy of 96.30% in comparison to the Toğaçar et al. model accuracy of 99.51%, similarly because no image augmentation and feature selection technique was used, whereas Toğaçar et al. [13] used the mRMR technique with better results. Furthermore, Jung et al. research team developed a three-dimensional ensemble CNN which required more training data and computational power to run [15].

Lastly, in a comparison of the final research group Lyu and Ling [12,16] in their multi-level CNN classification of lung nodules had a lower model performance accuracy of 84.41% mainly because no image augmentation nor feature selection method was used in their CNN architecture compared to the mRMR used by Toğaçar et al. [13].

Numerous studies assessing the performance of radiologists in lung nodule detection show low inter-observer agreement, varying sensitivities ranging from 30 to 97%, and false positive counts of 0.6-2.1 per patient, depending on the input data, method and criteria for identification [12]. A study from the NLST assessed CAD retrospectively in 134 subjects and found an improved inter-observer agreement (kappa increase from 0.53-0.66): results confirmed by similar studies [12]. As reducing inter-observer variation, one of the greatest advantages of CAD remains the detection of smaller lung nodules that are easily missed by radiologists/physicians [11]. The use of CAD by two radiologists in an emergency clinic study, did find improved reading time when CAD was used (Radiologist 1, 94.6 s vs. 102.7 s, P > 0.05; Radiologist 2, 61.1 s vs. 76.5 s, P < 0.05). Although this decrease in reading time was not statistically significant for both radiologists, they did get a significantly improved rate of nodule detection: 34% and 27% for Radiologists 1 and 2 respectively when CAD was reviewed after the CT images, but not when it was reviewed before the scans [10].

An observer performance study compared the performances of 10 radiologists without and with the use of CAD, in 50 CT examination cases [5]. Alternative free-response ROC curves for each output (with and without CAD) were calculated by plotting the true-positive fraction against the likelihood of obtaining an image with false-positive findings (i.e., with one or more false-positive lesions) at each confidence level. Using the area under each alternative free-response ROC curve (Az) to compare the observers’ performances, they found that the performance of all observers was significantly improved with the use of CAD (Table 1).

**Table 1:** Az values for performance in detecting all nodules. From Awai et al. (2003) [5].

Observer No.	Without CAD Output	With CAD Output
Board-certified radiologists		
1	0.49	0.52
2	0.64	0.70
3	0.74	0.79
4	0.59	0.61
5	0.66	0.67
Radiology residents		
6	0.65	0.70
7	0.70	0.76
8	0.56	0.57
9	0.75	0.78
10	0.64	0.65

Routine use of CAD by radiologists and physicians, especially in high-pressure environments, is justified due to improved rates of lung nodule detection, inter-observer agreement, interpretation speed, higher true-positive to false-positive ratios and for detection of small (<5 mm) nodules. The experiment conducted here performs well (Table 2), but it uses a small dataset. It may not perform well on a large production scale.

**Table 2:** Comparison of AI experimental models to detect lung cancer. [13-16].

Experiment	Performance Metric	Justification
Minimum redundancy, maximum relevance feature selection method on chest CT images with convolutional neural networks [13]	99.51%	CNN in conjunction with additional techniques: Image augmentation, PCA, mRMR and appropriate feature selection.
Convolutional neural network (CNN)-based PSO for lung nodule, false positive reduction of CT images [14]	97.62%	No image augmentation. No feature selection techniques before CNN.
Classification of lung nodules in CT scans using three-dimensional deep CNNs with a checkpoint ensemble method [15]	96.30%	No image augmentation. No feature selection techniques before CNN. Uses an ensemble with 3D: requiring more training datasets
Multi-level CNN for classification of lung nodules on CT images [16]	84.81%	No image augmentation. No feature selection techniques before CNN.

## Conclusions

Testing the model with larger datasets is needed to ensure they work on large, real production data. The image augmentation method is used here to increase the number of images: these techniques may lead to model overfitting. The KNN algorithm, which relies on the nearest neighbor, performed best on this dataset. It would be beneficial to further test these models on a new larger and different dataset. Lastly, the test dataset should not undergo image augmentation, but be tested from the original large dataset.
